# A Meta-Analysis of a Cohort Study on the Association between Sleep Duration and Type 2 Diabetes Mellitus

**DOI:** 10.1155/2021/8861038

**Published:** 2021-03-24

**Authors:** Huapeng Lu, Qinling Yang, Fang Tian, Yi Lyu, Hairong He, Xia Xin, Xuemei Zheng

**Affiliations:** ^1^Department of Hepatobiliary and Pancreas Surgery, The First Affiliated Hospital, Xi'an Jiaotong University, Xi'an, Shaanxi 710061, China; ^2^School of Nursing, Yan'an University, Yan'an, Shaanxi 710061, China; ^3^Department of Clinical Research Center, The First Affiliated Hospital, Xi'an Jiaotong University, Xi'an, Shaanxi, China; ^4^Department of Nursing, The First Affiliated Hospital, Xi'an Jiaotong University, Xi'an, Shaanxi 710061, China

## Abstract

**Objective:**

To study the association between sleep duration and the incidence of type 2 diabetes mellitus (T2DM) and to provide a theoretical basis for the prevention of T2DM through a meta-analysis.

**Methods:**

PubMed, Web of Science, Scopus, Embase, Cochrane Library, ProQuest, CNKI, Wanfang, VIP, and SINOMED were searched from their inception until May 2020. All cohort studies on the relationship between sleep duration and T2DM in adults were included. According to the inclusion and exclusion criteria, two authors independently assessed the literature and extracted the data. Metaregression and publication bias were evaluated, and sensitivity and meta-analyses were conducted with RevMan 5.3.

**Results:**

A total of 17 studies were collected, involving 737002 adults. The incidence of T2DM was 4.73% in short sleep duration (SSD) (*t* ≤ 6 h), 4.39% in normal sleep duration (NSD) (6 h < *t* < 9 h), and 4.99% in long sleep duration (LSD) (*t* ≥ 9 h). The meta-analysis demonstrated that SSD increased the risk of T2DM compared with NSD (RR = 1.22, 95% CI: 1.15-1.29, *P* < 0.001), LSD increased the risk of T2DM compared with NSD (RR = 1.26, 95% CI: 1.15-1.39, *P* < 0.001), and the risk of T2DM has no significant difference between SSD and LSD (RR = 0.97, 95% CI: 0.89-1.05, *P* = 0.41). The sensitivity of each study was robust and the publication bias was weak.

**Conclusion:**

SSD or LSD can increase the risk of T2DM.

## 1. Introduction

Diabetes mellitus (DM) is an epidemic disease in recent years. According to the International Diabetes Federation Diabetes Atlas Ninth Edition published in 2019, it is estimated that 463 million adults (aged between 20 and 79 years) have DM worldwide, and the prevalence has reached 9.3%. This number is expected to reach 578 million (10.2%) by 2030 and 700 million (10.9%) by 2045 [1]. With the aging of the population and the change of lifestyle in China, DM has become an epidemic. The prevalence of DM has soared from 0.67% in 1980 to 10.4% in 2013 [2]. China has 116.4 million people with DM nowadays. In 2019, 0.83 million patients died of DM and complications in China [1].

DM is one of the leading causes of retinopathy, vascular disease, neuropathy, amputation, heart disease, kidney failure, and premature death [3]. There are many risk factors for type 2 diabetes mellitus (T2DM), such as genetic factors and an unhealthy lifestyle. Studies have shown that sleep quality and sleep duration are also risk factors for T2DM [4–6]. However, relevant research conclusions are inconsistent. Some systematic reviews have indicated that short sleep or long sleep are risk factors for T2DM in adult [7–10] and risk factors for gestational diabetes mellitus in pregnant women [11–13]. However, studies for adults have not been updated in time. Therefore, we have conducted a meta-analysis of cohort studies to exhibit the relationship between sleep duration and T2DM in adults to provide the basis for primary prevention of T2DM.

## 2. Methods

### 2.1. Literature Search

We followed the preferred reporting items for systematic reviews and meta-analyses (PRISMA) guidelines in conducting this systematic review and meta-analysis [14]. We performed a serial literature search for English and non-English papers from inception to May 2020. We have systemically searched the Web of Science Core Collection (Science Citation Index Expanded: 1900–present; Social Sciences Citation Index: 1900–present; Arts & Humanities Citation Index: 1975–present; Conference Proceedings Citation Index—Science: 1996–present; Conference Proceedings Citation Index—Social Science & Humanities: 1996–present; and Emerging Sources Citation Index: 2015–present), PubMed, Scopus, Embase, the Cochrane Library, China National Knowledge Infrastructure (CNKI), Wanfang, VIP database, and SINOMED from inception to the present. We used the Boolean logic with search terms including “sleep duration,” “sleep amount,” “length of sleep,” “diabetes,” “diabetes mellitus,” “cohort stud^∗^,” “concurrent stud^∗^,” and “cohort analy^∗^.” To search for all terms that begin with a word, enter the word followed by an asterisk.  Box 1 provides the detailed search strategy for the Web of Science.

### 2.2. Study Eligibility and Selection Criteria

Two authors independently determined study eligibility. Any differences in opinion about eligibility were resolved through another author as a third-party consensus. The inclusion criteria were as follows: (1) cohort studies on the relationship between sleep duration and T2DM in adults, (2) reported sleep duration, (3) diagnosed T2DM, and (4) published English and non-English papers. Studies were not included if (1) they are cross-sectional studies, case-control studies, case reports, and review commentaries; (2) they do not report the incidence of T2DM; (3) they have participants < 10; (4) the subjects were special groups, such as pregnant women and patients after organ transplantation; (5) sleep duration was not reported clearly; (6) they were a duplicate report; and (7) they reported incomplete data and the relevant data were not available.

### 2.3. Definition of Variables and Outcomes

DM was defined as fasting glucose over 126 mg/dl or 2-hour postprandial glucose (at a glucose tolerance test) over 200 mg/dl or if the subjects were on diabetic drugs or insulin medication. The number of hours of sleep was defined as the average length of their sleep in whole hours at night. The usual sleep duration was self-reported. Taking sleep duration 6 hours < *t* < 9 hours as a reference, it is categorized as short sleep duration (SSD) (*t* ≤ 6 hours), normal sleep duration (NSD) (6 hours < *t* < 9 hours), and long sleep duration (LSD) (*t* ≥ 9 hours).

### 2.4. Data Abstraction and Validity Assessment

Data were independently recruited from all included studies on a template adopted from the Cochrane collaboration [15]. For all studies, we extracted the first author, publication year, study design, study location, study period, population, number of T2DM, age, gender (male/female), and data acquisition.

### 2.5. Assessment of Risk Bias

The risk bias of the included studies was independently assessed by two authors. The cohort study was evaluated by the Newcastle-Ottawa Scale (NOS), which included eight items, categorized into three groups: the selection of study groups, comparability of groups, and ascertainment of outcome [16]. Each study will be evaluated by eight items, and high-quality choices were identified with a star. There are a maximum of one star for each high-quality item within the selection and outcome categories and a maximum of two stars for comparability.

### 2.6. Statistical Analysis

The meta-analyses were conducted using Review Manager software, version 5.3 (https://community.cochrane.org/help/tools-and-software/revman-5). Dichotomous outcomes eligible in each study are reported as a risk ratio (RR) with an estimated 95% confidence interval (CI). Continuous outcomes are shown as the weighted mean difference (WMD) with the 95% CI, which were calculated from the mean, standard deviation (SD), *P* value, and sample size in each study. Heterogeneity was assessed using Higgins *I*^2^, which evaluates the percentage of total variation across studies that were due to heterogeneity rather than by chance. Thus, if *I*^2^ > 50%, which was considered to reflect substantial heterogeneity, a random effect model was used. If *I*^2^ ≤ 50%, which was considered to reflect no heterogeneity, a fixed effect model was employed. The chi-square tests were also used to evaluate the heterogeneity: *P* < 0.1 indicates heterogeneity, while *P* > 0.1 indicates no heterogeneity. Based on clinical knowledge, the study location and study period were considered to be responsible for heterogeneity, and so, these parameters were set as covariates in the meta-regression. Funnel plots judged the publication biases, and a *P* < 0.05 was considered statistically significant [17].

### 2.7. IRB Approval

This meta-analysis study was approved by the institutional review board of the Department of Hepatobiliary and Pancreas Surgery, The First Affiliated Hospital, Xi'an Jiaotong University.

## 3. Results

### 3.1. Eligible Studies

**Figure 1:**
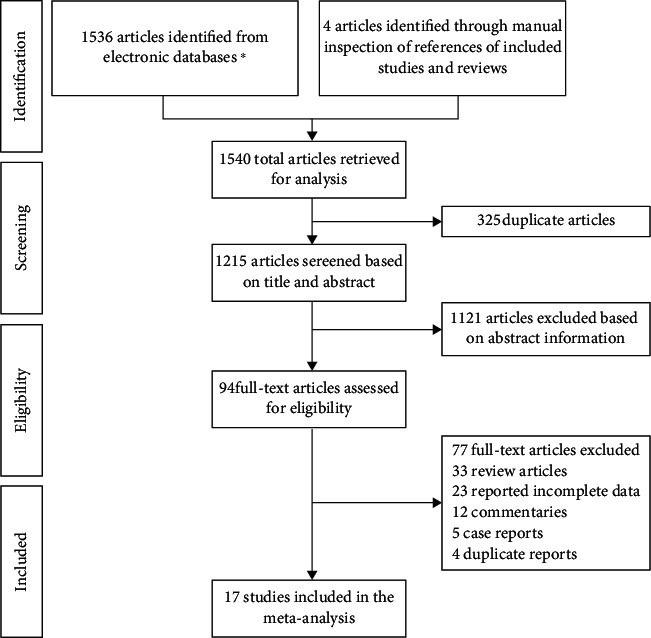
Summary of the literature identification and selection process. ∗ signifies Web of Science (442), PubMed (209), Scopus (490), Embase (117), Cochrane (71), ProQuest (34), CNKI (80), WanFang (61), VIP (10), and SinoMed (22).

A total of 1540 studies were identified, and 325 duplicated articles were excluded. One thousand one hundred and twenty-one studies were excluded after screening the title and abstract. After full-text screening, an additional 77 studies were excluded due to the following criteria: review article (*n* = 33), reported incomplete data (*n* = 23), commentary (*n* = 12), case report (*n* = 5), and duplicate report (*n* = 4). Finally, seventeen cohort studies were included in the present meta-analysis [18–34]. Among them, fifteen were in English [18–25, 27, 28, 30–34] and two were in Chinese [26, 29]. The flow chart of the study selection is summarized in Figure 1.

### 3.2. Study Characteristics

A total of 17 studies were selected for inclusion in this meta-analysis [18–34], with 737002 people. Among them were five each from China [21, 23, 25, 26, 29], and the United States [19, 28, 30, 31, 34], and one study each from Norway [18], South Korea [20], Britain [22], Australia [24], Japan [27], Finland [32], and Sweden [33]. Four studies' research subjects, those of Lou et al. [ 25]., Gao et al. [26], Li et al. [29], and Mallon et al. [33] were analyzed independently by gender. The research object of Ayas et al. is male [34]. A summary of the included studies is presented in Table 1.

### 3.3. Study Quality

The study quality for all 17 independent studies is shown in Table 2.

## 4. Meta-Analysis Results

### 4.1. A Meta-Analysis of the Relationship between SSD and T2DM

**Figure 2:**
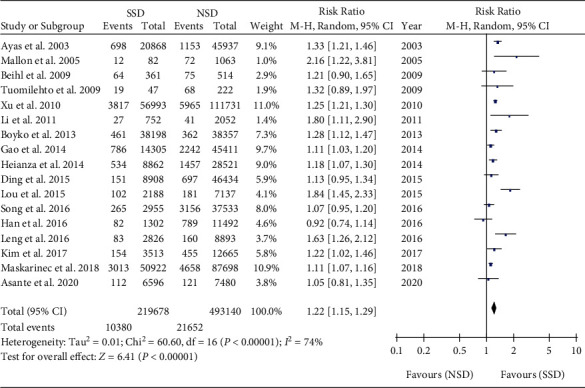
Pooled results for the association between SSD and T2DM.

We have analyzed a total of 17 studies that involved 712818 participants [18–34]. The incidence of T2DM was 4.73% (10380/219678) in SSD (*t* ≤ 6 h) and 4.39% (21652/493140) in NSD (6 h < *t* < 9 h). There was high heterogeneity among the studies, and the random effect model was used. Meta-analysis showed that the incidence of T2DM in SSD was higher than that in NSD (RR = 1.22, 95% CI: 1.15-1.29, *P* < 0.001) (Figure 2).

**Figure 3:**
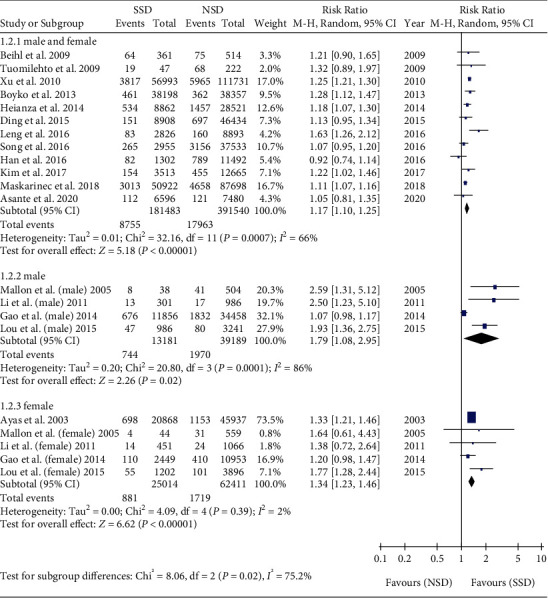
Gender subgroup analysis of the association between SSD and T2DM.

Subgroup analyses were performed among male [25, 26, 29, 33], female [25, 26, 29, 33, 34], and mixed male and female [18–24, 27, 28, 30–32]. Meta-analysis showed that the incidence of T2DM in SSD was higher than that in NSD in the male subgroup (RR = 1.79, 95% CI: 1.08-2.95, *P* = 0.02). Meta-analysis showed that the incidence of T2DM in SSD was higher than that in NSD in female subgroups (RR = 1.34, 95% CI: 1.23-1.46, *P* < 0.001). Meta-analysis showed that the incidence of T2DM in SSD was higher than that in NSD in the mixed male and female subgroup (RR = 1.17, 95% CI: 1.10-1.25, *P* < 0.001) (Figure 3).

**Figure 4:**
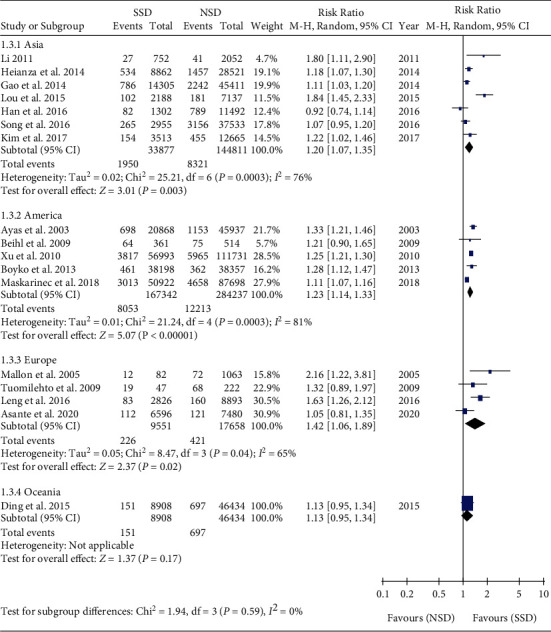
Regional subgroup analysis of the association between SSD and T2DM.

Subgroup analyses were performed among the Asia [20, 21, 23, 25–27, 29], America [19, 28, 30, 31, 34], Europe [18, 22, 32, 33], and Oceania subgroups [24]. Meta-analysis showed that the incidence of T2DM in SSD was higher than that in NSD in the Asia subgroup (RR = 1.20, 95% CI: 1.07-1.35, *P* = 0.003). Meta-analysis showed that the incidence of T2DM in SSD was higher than that in NSD in the America subgroups (RR = 1.23, 95% CI: 1.14-1.33, *P* < 0.001). Meta-analysis showed that the incidence of T2DM in SSD was higher than that in NSD in the Europe subgroup (RR = 1.42, 95% CI: 1.06-1.89, *P* = 0.02) (Figure 4).

### 4.2. Meta-Analysis of the Relationship between LSD and T2DM

**Figure 5:**
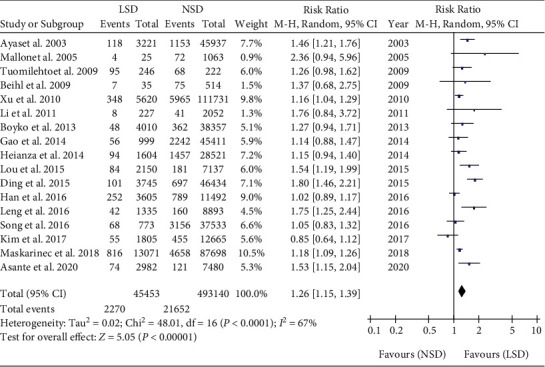
Pooled results for the association between LSD and T2DM.

We have analyzed a total of 17 studies that involved 538593 participants [18–34]. The incidence of T2DM was 4.99% (2270/45453) in LSD (*t* ≥ 9 h) and 4.39% (21652/493140) in NSD (6 h < *t* < 9 h). There was high heterogeneity among the studies, and the random effect model was used. Meta-analysis showed that the incidence of T2DM in LSD was higher than that in NSD (RR = 1.26, 95% CI: 1.15-1.39, *P* < 0.001) (Figure 5).

**Figure 6:**
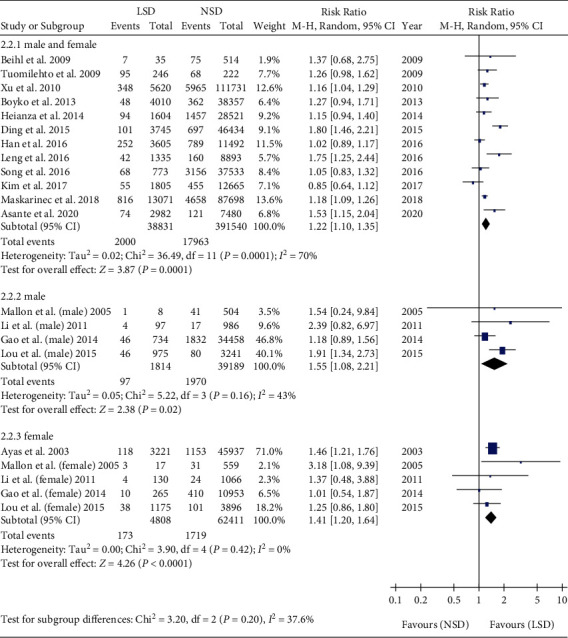
Gender subgroup analysis of the association between LSD and T2DM.

Subgroup analyses were performed among male [25, 26, 29, 33], female [25, 26, 29, 33, 34], and mixed male and female [18–24, 27, 28, 30–32]. Meta-analysis showed that the incidence of T2DM in LSD was higher than that in NSD in the male subgroup (RR = 1.55, 95% CI: 1.08-2.21, *P* = 0.02). Meta-analysis showed that the incidence of T2DM in LSD was higher than that in NSD in female subgroups (RR = 1.41, 95% CI: 1.20-1.64, *P* < 0.001). Meta-analysis showed that the incidence of T2DM in LSD was higher than that in NSD in the mixed male and female subgroup (RR = 1.22, 95% CI: 1.10-1.35, *P* = 0.0001) (Figure 6).

**Figure 7:**
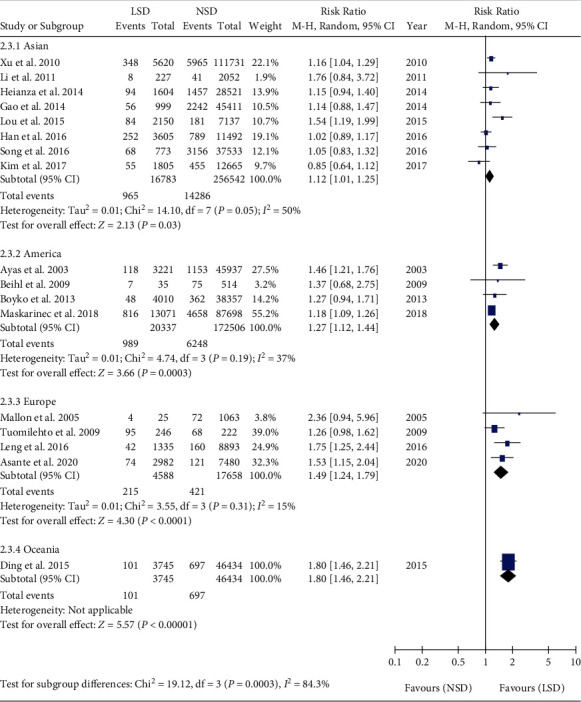
Regional subgroup analysis of the association between LSD and T2DM.

Subgroup analyses were performed among the Asia [20, 21, 23, 25–27, 29], America [19, 28, 30, 31, 34], Europe [18, 22, 32, 33], and Oceania subgroups [24]. Meta-analysis showed that the incidence of T2DM in LSD was higher than that in NSD in the Asia subgroup (RR = 1.12, 95% CI: 1.01-1.25, *P* = 0.03). Meta-analysis showed that the incidence of T2DM in LSD was higher than that in NSD in the America subgroups (RR = 1.27, 95% CI: 1.12-1.44, *P* = 0.0003). Meta-analysis showed that the incidence of T2DM in LSD was higher than that in NSD in the Europe subgroup (RR = 1.49, 95% CI: 1.24-1.79, *P* < 0.001) (Figure 7).

### 4.3. Meta-Analysis of SSD versus LSD for T2DM

**Figure 8:**
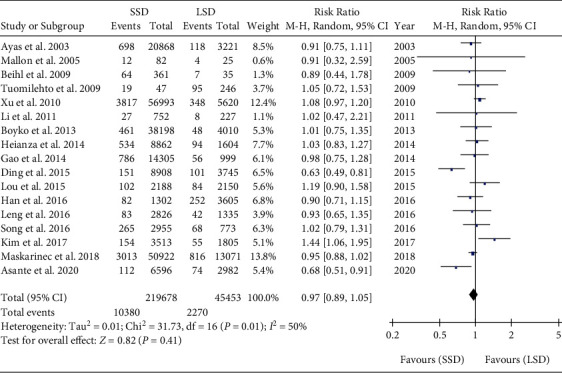
Pooled results of SSD versus LSD for T2DM.

There was high heterogeneity among the studies, and the random effect model was used. Meta-analysis showed that the incidence of T2DM was not significantly different between SSD and LSD (RR = 0.97, 95% CI: 0.89-1.05, *P* = 0.41) (Figure 8).

### 4.4. Sensitivity Analyses

**Figure 9:**
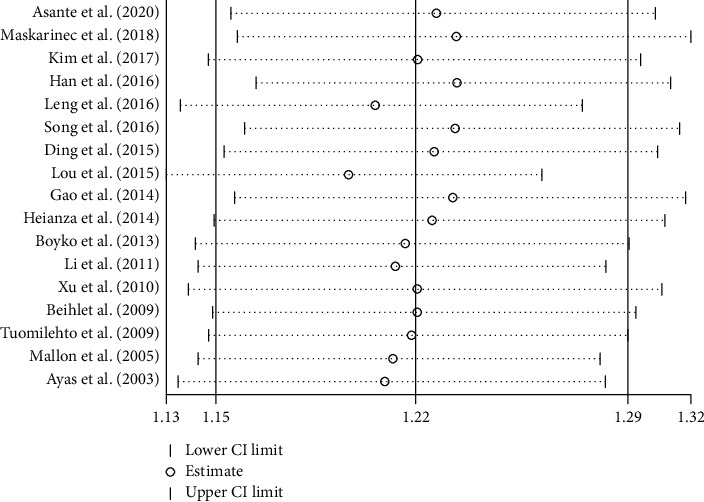
Sensitivity analyses of the association between SSD and T2DM.

**Figure 10:**
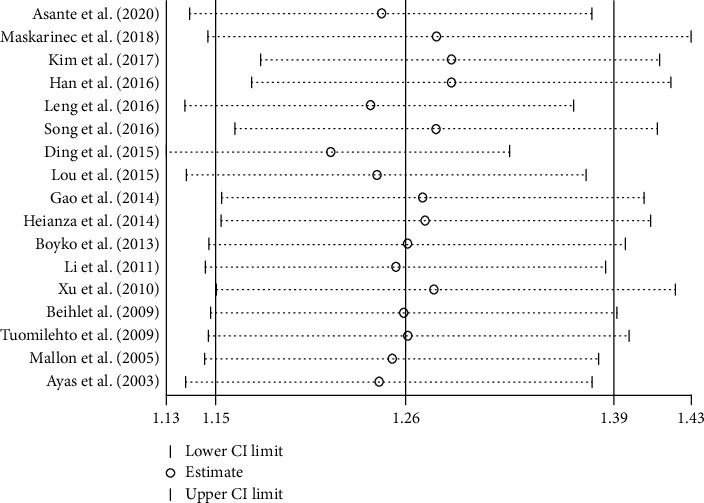
Sensitivity analyses of the association between LSD and T2DM.

Sensitivity analyses of the association between SSD, LSD, and T2DM were conducted. The results indicated that the sensitivity of the association between SSD and T2DM was robust after each study was excluded one by one. The RR was 1.22 and 95% CI was 1.15-1.29 (Figure 9). The results indicated that the sensitivity of the association between LSD and T2DM was robust after each study was excluded one by one. The RR was 1.26 and 95% CI was 1.15-1.39 (Figure 10). When the Chinese literature was excluded, the meta-analysis results and heterogeneity did not change significantly. However, according to gender subgroup analysis, the heterogeneity of the male subgroup and female subgroup decreased rapidly. The sensitivity of each study was robust.

### 4.5. Metaregression for the Sleep Duration and T2DM

Metaregression analyses were performed in the meta-analysis of the relationship between SSD and T2DM. The results indicated that neither the study location (coefficient = 0.021, SE = 0.050, *t* = 0.42, *P* = 0.678, 95% CI: −0.085 to 0.127) nor the study period (coefficient = 0.056, SE = 0.064, *t* = 0.87, *P* = 0.398, 95% CI: −0.082 to 0.194) was responsible for this heterogeneity. Other factors, such as age, cannot be fully extracted from the text.

Metaregression analyses were performed in the meta-analysis of the relationship between LSD and T2DM. Metaregression indicated that study location may have been responsible for this heterogeneity (coefficient = 0.154, SE = 0.040, *t* = 3.83, *P* = 0.002, 95% CI: 0.068 to 0.239). And no significant association was observed in the study period (coefficient = 0.135, SE = 0.075, *t* = 1.79, *P* = 0.095, 95% CI: −0.027 to 0.297).

### 4.6. Publication Bias Analyses

**Figure 11:**
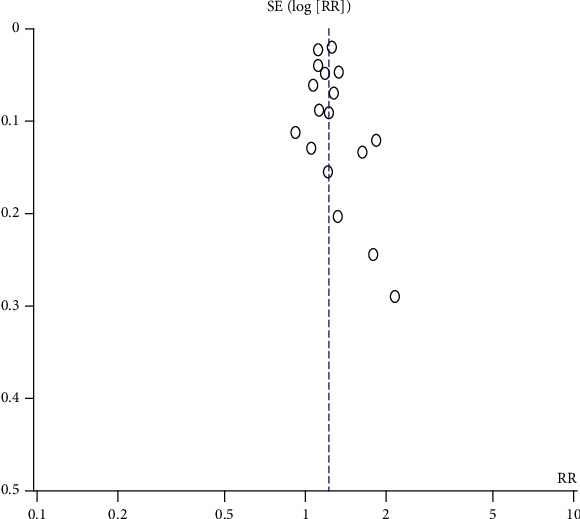
Funnel plots of publication bias for the association between SSD and T2DM.

**Figure 12:**
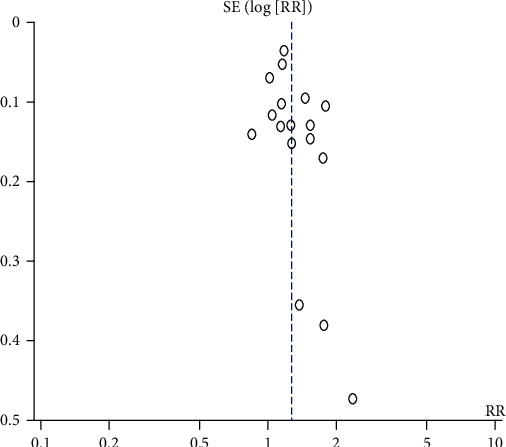
Funnel plots of publication bias for the association between LSD and T2DM.

Funnel plots of publication bias for the association between SSD, LSD, and T2DM were assessed. The symmetry found in the funnel plots indicated that the publication bias was weak. (Figures 11 and 12) Most of the studies are at the top of the funnel plot, indicating that the quality of the studies is good.

## 5. Discussion

The countries with the largest numbers of adults with DM aged 20–79 years in 2019 are China, India, and America with 116.4 million, 77 million, and 31 million DM patients (aged 20–79 years), respectively, and are anticipated to remain so in 2030 [1]. T2DM is closely related to unhealthy lifestyles [2]. Studies have shown that sleep is closely related to endocrine and metabolism [35]. With the progress of society and the development of the economy, the pace of modern life is getting faster and faster. Sleep discomfort causes many health problems. Studies have found that the average American sleep duration is from 9 hours in 1910 to 6.8 hours in 2005 [36]. Sleep discomfort causes not only endocrine disorders and other pathological states but also affects personal mental health. Therefore, improving sleep is a good measure to improve the quality of life.

In this meta-analysis of the association between SSD and T2DM, the risk of T2DM with SSD was 1.22 times higher than that with NSD, which was consistent with the conclusions of other cohort studies [37–45]. The risk of T2DM in male with SSD was 1.79 times higher than that with NSD. The risk of T2DM in females with SSD was 1.34 times higher than that with NSD. The risk of T2DM in males with SSD was slightly higher than that in females. Subgroup analysis by region showed that there was no significant difference among the Asian, American, and European subgroups. The risk of T2DM in Asia with SSD was 1.79 times higher than that with NSD. The risk of T2DM in America with SSD was 1.34 times higher than that with NSD. The risk of T2DM in Europe with SSD was 1.24 times higher than that with NSD. The mechanism of SSD leading to T2DM is still unclear. The possible reason is that sleep deprivation causes the imbalance of the sympathetic vagus nerve, which leads to the decrease of *β* cell response ability, the inhibition of insulin secretion, and the further development of insulin resistance and T2DM. Lack of sleep may also cause the release of a large number of inflammatory factors, inhibits the activity of islet receptor L-arginine kinase, and leads to insulin resistance. Some studies found that slow-wave sleep duration decreased insulin sensitivity and increased the risk of T2DM [46–48].

In this meta-analysis of the association between LSD and T2DM, the risk of T2DM with LSD was 1.26 times higher than that with NSD, which was consistent with the conclusions of other cohort studies [37–45]. The risk of T2DM in male with LSD was 1.55 times higher than that with NSD. The risk of T2DM in females with LSD was 1.27 times higher than that with NSD. Subgroup analysis by region showed that there were differences among the Asian, American, and European subgroups. The risk of T2DM in Asia with LSD was 1.12 times higher than that with NSD. The risk of T2DM in America with LSD was 1.27 times higher than that with NSD. The risk of T2DM in Europe with LSD was 1.49 times higher than that with NSD. There are few studies on the relationship between LSD and T2DM. The reason may be that long sleeps are a poor sleep quality actually and they may prolong sleep duration to make up for the impact of poor sleep quality. Sleeping for a long time is harmful to health itself. Excessive sleep duration may be associated with other factors, such as obesity, poor social and economic status, low physical activity, depression, sleep apnea, or other chronic diseases. These factors can produce a confounding effect [46, 47, 49].

The meta-analysis found that LSD or SSD will increase the risk of T2DM. Some studies suggest that LSD or SSD can lead to impaired fasting blood glucose, abnormal HbA1, and insulin resistance [50–56]. There was a U-shaped dose response relationship between sleep duration and risk of T2DM [10, 57]. SSD or LSD increased the risk of T2DM. Therefore, the risk of T2DM can be reduced if the sleep duration of the patients with poor sleep is changed and the sleep duration is maintained between 7 and 8 hours. SSD or LSD is a risk factor for chronic diseases such as DM, coronary heart disease, stroke, and obesity [7, 9, 58, 59]. The sensitivity of each study was robust and the publication bias was weak. While we found that the study location may have been responsible for this heterogeneity in the meta-analysis of the relationship between LSD and T2DM, other factors, such as age, cannot be fully extracted from the text.

### 5.1. Limitations

Limitations are listed as follows: (1) some studies have not been retrieved. All the included studies are published in Chinese and English, and there may be incomplete literature retrieval, (2) the overall heterogeneity of the study is high, suggesting that there is heterogeneity among the studies. However, after subgroup analyses were performed, the heterogeneity of subgroups decreased significantly, (3) the study explored the association between sleep duration and T2DM but paid less attention to the association between sleep quality and T2DM, which also indicated that we should focus on it in later research, and (4) for special populations such as gestational diabetes, postpartum diabetes, and posttransplant diabetes, there is less attention.

More and more studies have confirmed that an unhealthy lifestyle plays an important role in the pathogenesis of T2DM and glucose control in DM [60]. From the perspective of prevention, appropriate sleep duration can be used as the primary prevention of T2DM [7, 9, 61, 62]. Good sleep should be considered as an important health component in the prevention and treatment of T2DM.

## 6. Conclusions

The purpose of the current study was to determine the cohort studies of the relationship between sleep duration and T2DM in adults with a systematic review and meta-analysis. This study provides the first systematic assessment of the cohort study of the relationship between sleep duration and T2DM. The findings indicate that SSD or LSD is a risk factor for T2DM. The findings of this study have several important implications for future practice. Further research is required to attend to the association between sleep quality and T2DM.

## Figures and Tables

**Box 1:**
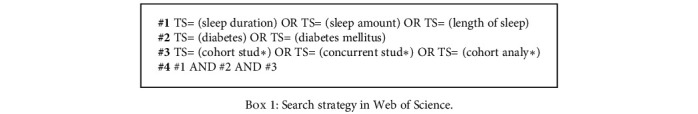
Search strategy in Web of Science.

**Table 1:** Characteristics of included studies.

Study	Study design	Study location	Study period	Total	T2DM	Age	Gender (male/female)	Data acquisition
Asante et al. (2020) [18]	Prospective cohort study	Nord-Trøndelag, Norway	2006–2008	17058	362	20–55	NR	Questionnaires, laboratory measurements
Maskarinec et al. (2018) [19]	Prospective cohort study	Hawaii and California, USA	1993–1996	151691	8487	45–75	69097/82594	Self-reported
Kim et al. (2017) [20]	Cohort study	Seoul and Suwon, South Korea	2011	17983	664	41 ± 5.9	11995/5988	Questionnaires
Han et al. (2016) [21]	Cohort study	Hubei, China	2008–2013	16399	1123	62.5	7083/9316	Face-to-face interviews
Leng et al. (2016) [22]	Prospective cohort study	Britain	1998–2000	13465	285	40–74	5887/7578	Questionnaires
Song et al. (2016) [23]	Prospective cohort study	Hebei, China	2006.6–2015	56588	4899	49	43494/13094	Face-to-face interviews
Ding et al. (2015) [24]	Cohort study	New South Wales, Australia	2006–2010	54997	888	≥45	25038/29959	Self-reported
Lou et al. (2015) [25]	Prospective cohort study	Xuzhou, China	2008–2013	11842	367	≥18	5375/6467	Interviews, laboratory measurements
Gao et al. (2014) [26]	Prospective cohort study	Hebei, China	2006–2011	60715	3084	49.14 ± 11.96	47048/13667	Questionnaires
Heianza et al. (2014) [27]	Prospective cohort study	Niigata, Japan	1999.4–2004.3	38987	2085	18–83	NR	Questionnaires
Boyko et al. (2013) [28]	Cohort study	USA	2001–2007	47093	871	34.9 ± 9.0	35037/12056	Self-reported, questionnaires
Li et al. (2011) [29]	Cohort study	Jiangsu, China	2004–2007	3031	76	52.28 ± 12.31	1381/1647	Questionnaires
Xu et al. (2010) [30]	Prospective cohort study	USA	2000–2006	174542	10143	50–71	99060/75284	Questionnaires
Beihl et al. (2009) [31]	Cohort study	USA	1992.10–1999.7	900	146	40–69	390/510	Interviews
Tuomilehto et al. (2009) [32]	Prospective cohort study	Finland	2004.10–2005.1	515	182	40–64	170/345	Interviews, laboratory measurements
Mallon et al. (2005) [33]	Cohort study	Sweden	1983–1995	1170	88	45–65	550/620	Questionnaire
Ayas et al. (2003) [34]	Cohort study	USA	1986–1996	70026	1969	30–55	0/70026	Interviews

NR: not reported.

**Table 2:** Quality of included studies.

Study	Selection				Comparability		Outcome		
	1	2	3	4	5A	5B	6	7	8
Asante et al. (2020) [18]	Y	Y	Y	Y	Y	Y	Y	Y	Y
Maskarinec et al. (2018) [19]	Y	Y	Y	Y	Y	Y	Y	Y	Y
Kim et al. (2017) [20]	Y	Y	Y	Y	Y	Y	Y	Y	Y
Han et al. (2016) [21]	Y	Y	Y	Y	Y	Y	Y	Y	N
Leng et al. (2016) [22]	Y	Y	Y	Y	Y	Y	Y	Y	N
Song et al. (2016) [23]	N	Y	Y	Y	Y	Y	Y	Y	Y
Ding et al. (2015) [24]	Y	Y	Y	Y	Y	Y	Y	Y	Y
Lou et al. (2015) [25]	Y	Y	Y	Y	Y	Y	Y	Y	Y
Gao et al. (2014) [26]	Y	Y	Y	Y	N	N	Y	Y	N
Heianza et al. (2014) [27]	Y	Y	Y	Y	Y	Y	Y	Y	N
Boyko et al. (2013) [28]	N	Y	Y	Y	Y	Y	Y	Y	N
Li et al. (2011) [29]	Y	Y	Y	Y	Y	Y	Y	Y	Y
Xu et al. (2010) [30]	Y	Y	Y	Y	Y	Y	Y	Y	N
Beihl et al. (2009) [31]	Y	Y	Y	Y	Y	Y	Y	Y	N
Tuomilehto et al. (2009) [32]	N	Y	Y	Y	Y	Y	Y	Y	N
Mallon et al. (2005) [33]	Y	Y	Y	Y	Y	Y	Y	Y	Y
Ayas et al. (2003) [34]	Y	Y	Y	Y	Y	Y	Y	Y	N

Y: yes; N: no.

## Data Availability

The data used to support the findings of this study are included within the article.
